# D-Psicose mitigates NAFLD mice induced by a high-fat diet by reducing lipid accumulation, inflammation, and oxidative stress

**DOI:** 10.3389/fnut.2025.1574151

**Published:** 2025-05-27

**Authors:** Jiajun Tan, Wen Sun, Xueyun Dong, Jiayuan He, Asmaa Ali, Min Chen, Leilei Zhang, Liang Wu, Keke Shao

**Affiliations:** ^1^Department of Laboratory Medicine, School of Medicine, Jiangsu University, Zhenjiang, China; ^2^Department of Laboratory Medicine, The Yancheng Clinical College of Xuzhou Medical University, The First People's Hospital of Yancheng, Yancheng, China; ^3^Critical Care Medicine, Jurong Hospital Affiliated to Jiangsu University, Zhenjiang, China; ^4^Health Testing Center, Zhenjiang Center for Disease Control and Prevention, Zhenjiang, China; ^5^Department of Pulmonary Medicine, Abbassia Chest Hospital, EMOH, Cairo, Egypt; ^6^Public Experiment and Service Center, Jiangsu University, Zhenjiang, China; ^7^Molecular Medical Research Center, Yancheng Clinical Medical College of Jiangsu University, Yancheng, China

**Keywords:** D-Psicose, non-alcoholic fatty liver disease, oxidative stress, gut microbiota, metabolomics

## Abstract

D-Psicose (DPS) serves as an optimal sucrose substitute, providing only 0.3% of sucrose’s energy content, while exhibiting anti-inflammatory properties and inhibiting lipid synthesis. However, its efficacy in managing non-alcoholic fatty liver disease (NAFLD) remains unclear. This study employed network pharmacology and molecular docking to identify potential DPS targets for NAFLD treatment. A high-fat diet was used to induce a NAFLD mouse model, with DPS administered in drinking water at 5% (high dose DPS group, DPSH group) and 2.5% (low dose DPS group, DPSL group) concentrations. After 12 weeks, blood lipid levels, liver lipid deposition, and inflammation were evaluated to assess the therapeutic effects of DPS. To explore its underlying mechanisms, colon contents 16S rRNA sequencing and serum untargeted metabolomics were performed. Results indicated that DPS significantly reduced lipid accumulation and inflammatory damage in the livers of NAFLD mice, improving both blood lipid profiles and oxidative stress. Network pharmacology analysis revealed that DPS primarily targets pathways associated with inflammation and oxidative stress, while molecular docking suggested its potential to inhibit the NF-κB pathway activation and the expression of the receptor for advanced glycation end-products (RAGE), findings corroborated by Western blotting. Additionally, gut microbiota and serum metabolomics analyses demonstrated that DPS improved microbiota composition by increasing the abundance of beneficial bacteria, such as *Akkermansia*, and restored serum metabolomic balance, enhancing anti-inflammatory and antioxidant metabolites like Tretinoin and Pyridoxamine. The non-targeted metabolomics results suggest that DPS is mediated by glutathione metabolism, arginine and proline metabolism, unsaturated fatty acid biosynthesis, and linoleic acid metabolism interferes with NAFLD progression. In conclusion, DPS may alleviate oxidative stress and lipid accumulation in NAFLD mice through the AGEs/RAGE/NF-κB pathway, while also ameliorating gut microbiota dysbiosis and serum metabolomic disturbances, fostering the production of anti-inflammatory and antioxidant metabolites.

## 1 Introduction

D-Psicose (DPS), a ketohexose monosaccharide and epimer of D-fructose at the C-3 position, is found in trace amounts in wheat, itea plants, and processed cane and beet molasses, and can be bioengineered from fruit and vegetable waste (1, 2). With 70% of the sweetness of sucrose but only 0.3% of its caloric content, DPS presents an ideal sucrose alternative (3–5). It offers several health benefits, including anti-inflammatory, antioxidant, glucose and lipid metabolism regulation, and neuroprotective effects (6–8). Animal studies suggest DPS can inhibit liver fat-producing enzymes and intestinal *α*-glucosidase, potentially reducing body fat accumulation (9, 10). However, its therapeutic potential for non-alcoholic fatty liver disease (NAFLD) remains underexplored.

NAFLD affects approximately 25% of the global population and is a major contributor to cirrhosis and hepatocellular carcinoma (11, 12). The disease progresses from steatosis, with or without mild inflammation, to non-alcoholic steatohepatitis (NASH), leading to significant necroinflammation and accelerated fibrosis compared to simple fatty liver (13–15). NAFLD is closely associated with metabolic syndrome, with type 2 diabetes significantly increasing the risk of cirrhosis and its complications (16–18). Currently, no specific treatments for NAFLD have been approved (13, 19–21). The recent FDA approval of Resmetirom (Rezdiffra™) in March 2024 marked a milestone as the first drug specifically indicated for non-cirrhotic NASH with moderate-to-advanced fibrosis (22). However, its clinical adoption faces notable limitations including diarrhea, nausea, and transient elevations in LDL cholesterol, posing risks for long-term use in metabolically compromised populations (23). Existing strategies emphasize the importance of lifestyle modifications and weight loss for both prevention and management (24–26). Leveraging DPS’s low-calorie and anti-inflammatory properties, network pharmacology, molecular docking, and an NAFLD mouse model were employed to investigate its therapeutic effects. Additionally, gut microbiota 16S rRNA sequencing and serum untargeted metabolomics were utilized to uncover the underlying mechanisms of DPS in NAFLD treatment, presenting a novel therapeutic approach.

## 2 Materials and methods

### 2.1 Network pharmacology and molecular docking analysis of DPS in the treatment of NAFLD

The DPS structure, obtained from the PubChem database, was imported into the SwissTarget Prediction and Superpred databases to predict potential targets. Searches were conducted in the OMIM^1^ and GeneCards^2^ databases using the keyword “Nonalcoholic fatty liver disease.” A Venn diagram was generated using the VennDiagram package in R Studio^3^ to display the overlapping potential targets of DPS for NAFLD treatment. The identified targets were imported into the STRING database, with “*Homo sapiens*” specified as the species. The results were saved and imported into Cytoscape 3.10.0 for visualization, where the CytoHubba plugin was employed to rank the top 10 key targets by “Degree.”

The potential targets were uploaded to the DAVID database^4^ for GO functional enrichment and KEGG pathway analyses, applying a significance threshold of *p* < 0.05 to identify relevant signaling pathways associated with DPS treatment of NAFLD. The ggplot2 package in R Studio was used to generate bar charts for the top 10 signaling pathways in the biological process (BP), cellular component (CC), and molecular function (MF) GO enrichment categories. Additionally, the top 15 KEGG pathway results were visualized as a bubble chart.

The 3D structures of DPS active components were retrieved from the PubChem database as small molecule ligands. Core target proteins with high degree values in the PPI network were selected as receptor proteins, and their 3D structures were obtained from the PDB database.^5^ PyMOL software was utilized to prepare receptor proteins by adding hydrogen atoms, removing water molecules, and eliminating small molecule ligands. AutoDockTools was used to validate the molecular docking of key active components with core target proteins, and PyMOL was used for visualizing the results with enhanced activity.

### 2.2 Experimental animals and grouping

Eight-week-old male institute of cancer research (ICR) mice, purchased from Wukong Biotechnology (Nanjing, China), were housed at Jiangsu University’s Experimental Animal Center. The mice were maintained under controlled conditions (25°C, 50% relative humidity, 12 h light/dark cycle). They were randomly assigned to four groups: normal control (NC, *n* = 6), non-alcoholic fatty liver disease (NAFLD, *n* = 6), low-dose DPS (DPSL, *n* = 6), and high-dose DPS (DPSH, *n* = 6). The NC group received a standard diet, while the NAFLD, DPSL, and DPSH groups were fed a high-fat diet to induce NAFLD, following the method outlined by Sun et al. (27). The DPSL and DPSH groups received DPS in drinking water at concentrations of 2.5 and 5%, respectively, for 12 weeks. At the end of the treatment period, the mice were euthanized with an intraperitoneal injection of urethane (700 mg/kg; Sigma-Aldrich, St. Louis, MO, United States), and serum, liver, and colonic content were collected.

### 2.3 Analysis of serum biochemical markers

According to He et al. (28), kits from Nanjing Jiancheng Bioengineering Institute (Nanjing, China) were used to measure the concentrations and activities of alanine aminotransferase (ALT, C009-2-1), aspartate aminotransferase (AST, C010-2-1), triglycerides (TG, A110-1-1), total cholesterol (TC, A111-1-1), low-density lipoprotein cholesterol (LDL-C, A113-1-1), high-density lipoprotein cholesterol (HDL-C, A112-1-1), malondialdehyde (MDA, A003-1-2), and superoxide dismutase (SOD, A001-3-2) in mouse serum. Testing procedures followed the kit manuals’ instructions.

### 2.4 Analysis of hepatic inflammatory factor expression

The expression levels of inflammatory factors in mouse liver, including TNF-*α*, IL-1β, IL-10, NLRP3, and Caspase-1 mRNA, were measured using the qRT-PCR assay, as outlined by He et al. (28). Reagents for the qRT-PCR assay were provided by Vazyme Biotech Co., Ltd. (Nanjing, China). Primer sequences are listed in Supplementary Table S1.

### 2.5 Western blotting assay

The expression of the inflammation-related factor RAGE and the phosphorylation level of NF-κB p65 in the mouse liver were determined *via* Western blotting, with experimental procedures detailed in Supplement S2.

### 2.6 Histological analysis of mouse liver tissue using HE and Oil Red O staining

Liver damage was assessed using H&E staining, while lipid deposition in the liver was evaluated with Oil Red O staining. The staining procedures are described in Supplement S3.

### 2.7 Analysis of gut microbiota 16S rRNA sequencing

The procedure for microbial 16S rRNA analysis of mouse colon contents can be found in Supplement S4. Genomic DNA was extracted from mouse colon microbiota using a genomic DNA extraction kit (TIANGEN, Beijing, China) for subsequent gut microbiota analysis, conducted by Wekemo Tech Group Co., Ltd. (Shenzhen, China). Bioinformatics analysis facilitated sequencing and species identification. The *α*-diversity (shannon) and *β*-diversity (unweighted unifrac and bray curtis) of the gut microbiota in mice were applied to evaluate microbial composition similarity or dissimilarity between groups. A clustering heatmap was used to investigate the impact of DPS on the gut microbiota.

### 2.8 Analysis of untargeted serum metabolomics

The untargeted metabolomics analysis of mouse serum was performed as follows: 120 μL of precooled 50% methanol was mixed with 20 μL of serum, incubated at room temperature for 10 min, and stored overnight at −20°C. After centrifugation at 4000 × g for 20 min, the supernatant was collected for untargeted metabolomics analysis. This analysis was conducted by Wekemo Tech Group Co., Ltd. (Shenzhen, China). PCA was used to illustrate the differences in serum metabolites across groups, while OPLS-DA provided the variable importance in projection (VIP) and significance values (*p*-value) for metabolite differences between groups. Metabolites were considered significant if VIP > 1.0 and *p* < 0.05 and were uploaded to the MetaboAnalyst 6.0 platform for pathway analysis.

### 2.9 Statistical analysis

Data analysis was performed using SPSS 20.0 (SPSS, Chicago, IL, United States), with results presented as mean ± SD. One-way ANOVA and Tukey’s *post hoc* method was used to assess significant differences across groups, with *p* < 0.05 considered statistically significant. Graphs were generated using GraphPad Prism, the Bioincloud platform^6^, and R Studio.

## 3 Results

### 3.1 Network pharmacology analysis results of DPS in the treatment of NAFLD

**Figure 1:**
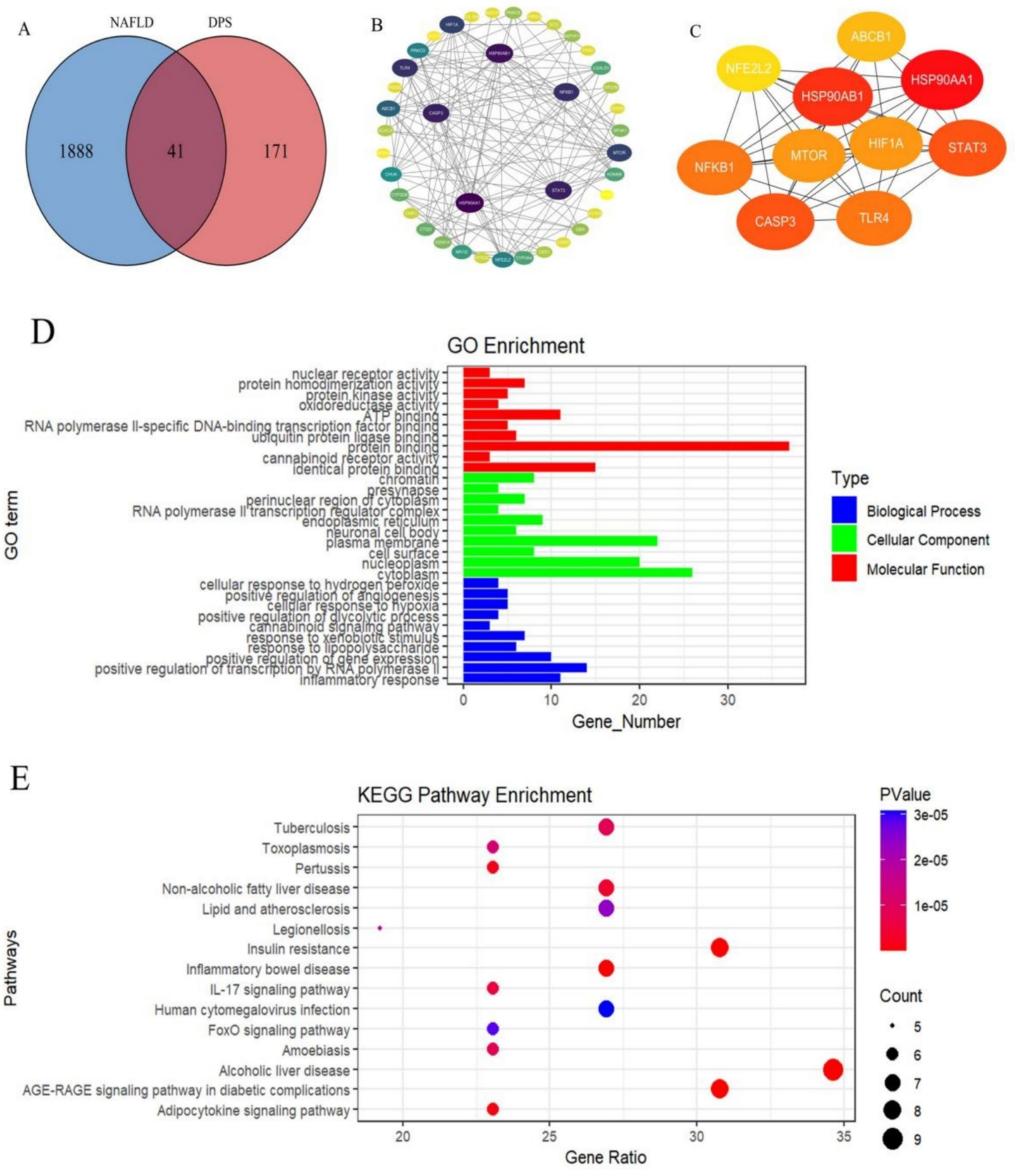
Using network pharmacology analysis to identify the targets of DPS in treating NAFLD. There were 41 target genes for DPS in treating NAFLD **(A)**. These 41 targets were input into the STRING database to obtain the PPI network **(B)**. A PPI network consisting of 40 nodes (representing functional proteins) and 146 edges (representing interactions between proteins) was constructed using Cytoscape software, and the top 10 hub genes in the PPI network were identified based on “Degree” **(C)**. Further, GO enrichment analysis of DPS treatment targets was performed using the DAVID database **(D)**, and the KEGG signaling pathways of DPS treatment targets for NAFLD were predicted **(E)**.

The SwissTargetPrediction and Targets SUPPERD databases were utilized to identify 212 potential targets related to DPS, while 1929 NAFLD-related targets were obtained from the GeneCards and OMIM databases (Figure 1A). Forty-one overlapping targets between DPS and NAFLD were identified as candidate genes for DPS treatment of NAFLD. These target genes were subsequently input into the STRING database to generate the protein–protein interaction (PPI) network (Figure 1B). A PPI network comprising 40 nodes (representing functional proteins) and 146 edges (depicting protein–protein interactions) was constructed using Cytoscape software. The top 10 hub genes, ranked by degree, were HSP90AA1, HSP90AB1, STAT3, CASP3, and NFKB1 (Figure 1C). These hub genes are likely key targets for DPS in the treatment of NAFLD. GO enrichment analysis was performed using the DAVID database, revealing 145 statistically significant GO terms. The analysis indicated that the DPS treatment targets were predominantly enriched in biological processes such as the inflammatory response, RNA polymerase II-mediated transcription regulation, gene expression enhancement, and lipopolysaccharide response. CCs were primarily enriched in the cytoplasm, nucleoplasm, cell surface, and plasma membrane. MFs of DPS targets were enriched in processes like identical protein binding, cannabinoid receptor activity, protein binding, and ubiquitin protein ligase binding (Figure 1D).

To further explore the mechanisms underlying DPS’s effects on NAFLD, KEGG pathway analysis of the DPS targets was performed using the DAVID database, identifying 56 statistically significant pathways. The top five pathways were Th17 cell differentiation, NOD-like receptor signaling, chemical carcinogenesis *via* receptor activation, lipid metabolism and atherosclerosis, and alcoholic liver disease (Figure 1E). These results suggest that DPS may exert its therapeutic effects by inhibiting inflammation and alleviating NAFLD symptoms through multiple pathways.

### 3.2 The molecular docking results of DPS with key therapeutic targets for NAFLD

**Figure 2:**
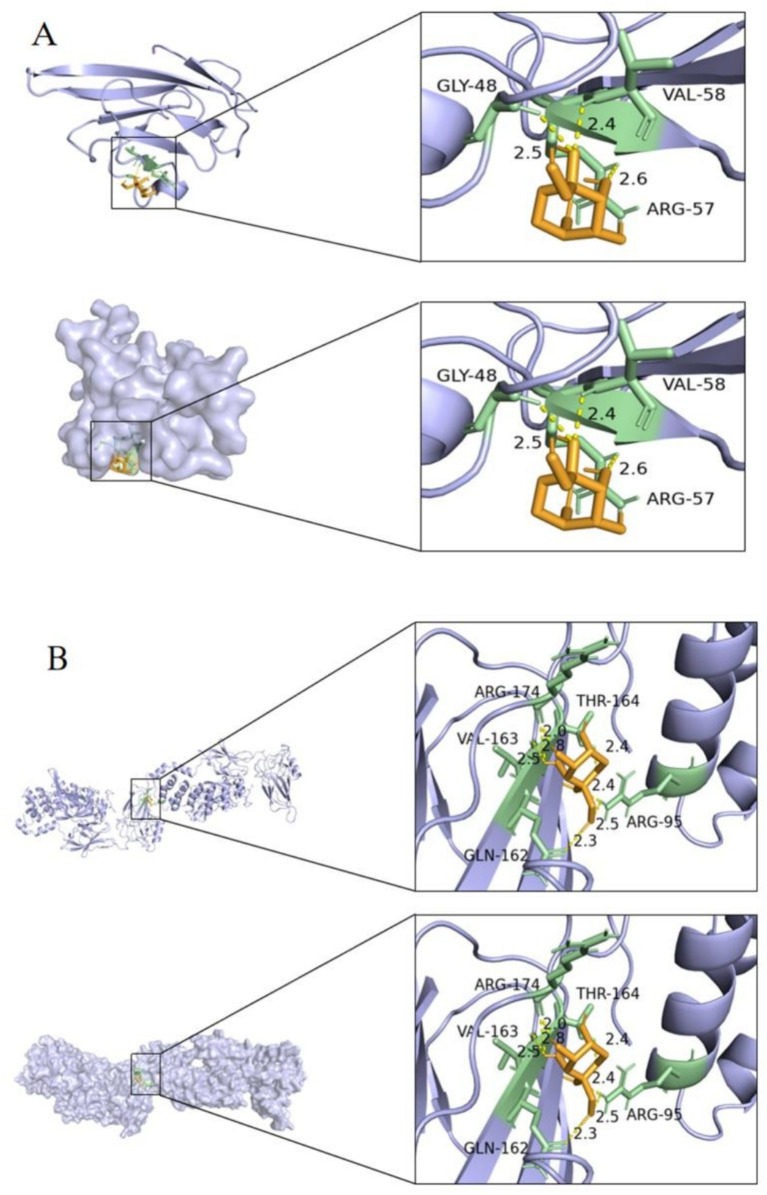
Diagram of the docking model between DPS and key targets. The minimum binding energy between DPS and RAGE was less than −20.50 kJ/mol **(A)**, and the minimum binding energy between DPS and NF-κB was less than −23.01 kJ/mol **(B)**. These results indicate that DPS has a good binding ability with both NF-κB and RAGE.

RAGE was selected as the receptor for molecular docking, based on its role as a hub target gene in the NF-κB pathway and its involvement in the AGE-RAGE signaling pathway, which is critical in diabetic complications. DPS was used as the ligand for molecular docking, with binding stability assessed by binding energy. A binding energy below −20.0 kJ/mol indicates strong molecular affinity with the protein. Molecular docking results showed that DPS forms three hydrogen bonds with RAGE (GLY-48, VAL-58, and ARG-57) and the lowest binding energy of −20.50 kJ/mol (Figure 2A). Additionally, DPS and NF-κB formed five hydrogen bonds (ARG-174, THR-164, VAL-163, ARG-95, and GLN-162), with a binding energy of −23.01 kJ/mol (Figure 2B). These results suggest that DPS exhibits strong binding affinity with both NF-κB and RAGE.

### 3.3 DPS reduced liver lipid accumulation and liver lesions in NAFLD mice

**Figure 3:**
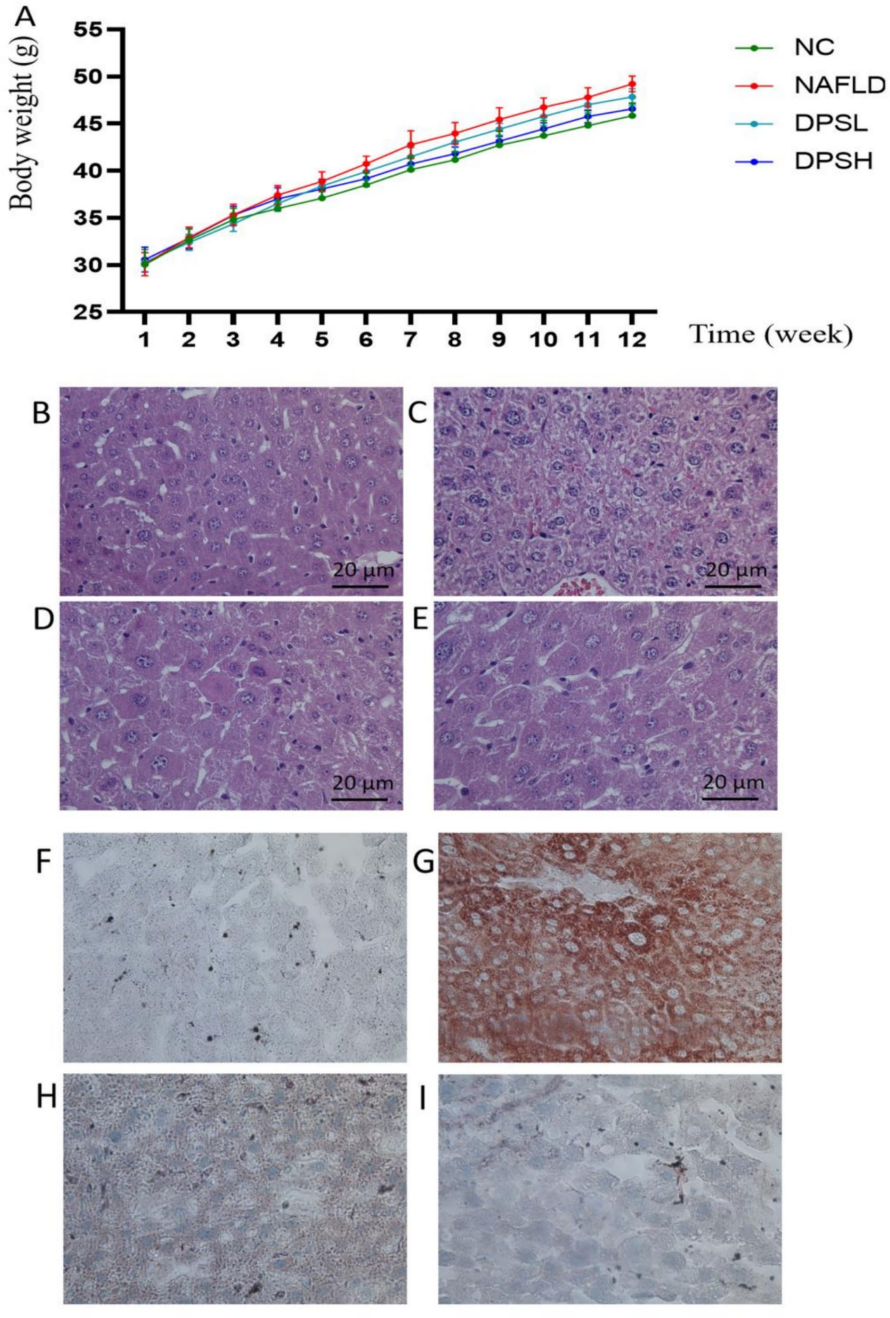
Body weight of mice in the experiment **(A)**. Liver damage in mice was observed using HE staining **(B–E)**, and lipid deposition in the liver was assessed using Oil Red O staining **(F–I)**. **(B,F)** The NC group; **(C,G)** The NAFLD group; **(D,H)** The DPSL group; **(E,I)** The DPSH group.

From week 6 onwards, the NAFLD group exhibited a significant increase in body weight compared to the NC group. No significant differences in body weight were observed between the NAFLD, DPSL, and DPSH groups during the experiment (*p* > 0.05) (Figure 3A). HE staining revealed a marked reduction in hepatocyte ballooning degeneration and necrosis in the DPSL and DPSH groups relative to the NAFLD group (Figures 3B–E). Oil Red O staining showed a significant reduction in hepatocyte fat deposition in the DPSL and DPSH groups (Figures 3F–I).

**Figure 4:**
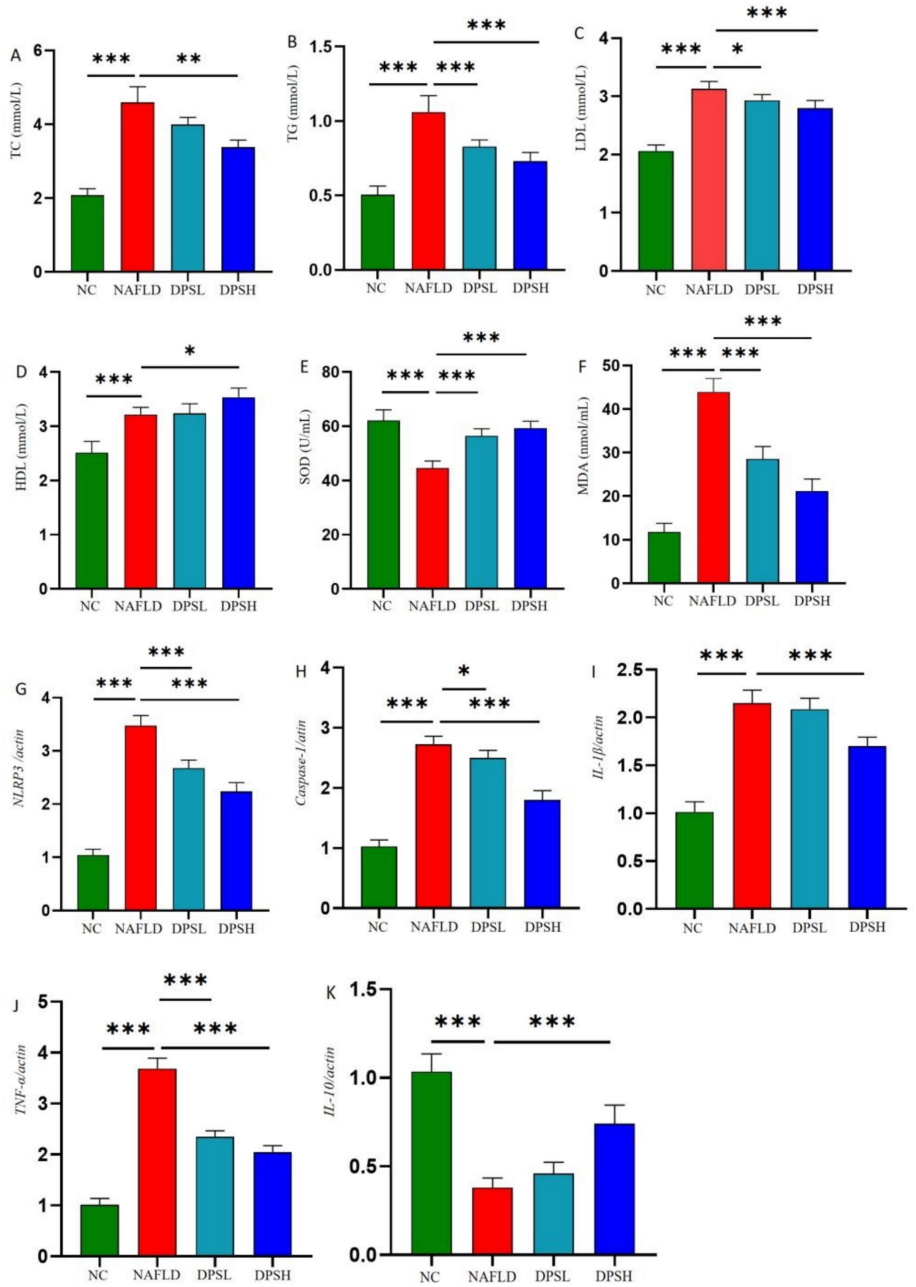
Levels of lipid-related indicators in mouse serum: TC **(A)**, TC **(B)**, LDL **(C)**, and HDL **(D)**; levels of oxidative stress-related indicators in serum: SOD **(E)** and MDA **(F)**; inflammatory factors in mouse liver: NLRP3 **(G)**, Caspase-1 **(H)**, IL-1β **(I)**, TNF-*α* **(J)**, and IL-10 **(K)**. *n* = 6. *: *p* < 0.05; **: *p* < 0.01; ***: *p* < 0.001.

Compared to the NAFLD group, the DPSL and DPSH groups exhibited significantly lower serum levels of TC, TG, and LDL-C, while the DPSH group demonstrated a significant increase in HDL-C levels (*p* < 0.05) (Figures 4A–D). Oxidative stress in serum was notably diminished, as indicated by a significant increase in SOD activity and a marked decrease in MDA levels (*p* < 0.05) (Figures 4E,F).

qPCR analysis of liver tissue showed that DPS significantly reduced hepatic expression of inflammatory factors. The DPSL and DPSH groups exhibited lower mRNA levels of *NLRP3*, *Caspase-1*, and *TNF-α* compared to the NAFLD group, with *IL-1β* also significantly reduced in the DPSH group (*p* < 0.05). Furthermore, the DPSH group demonstrated a significant increase in hepatic IL-10 mRNA expression (*p* < 0.05) (Figures 4G–K).

### 3.4 DPS reduced RAGE expression and NF-κB p65 protein phosphorylation in the liver

**Figure 5:**
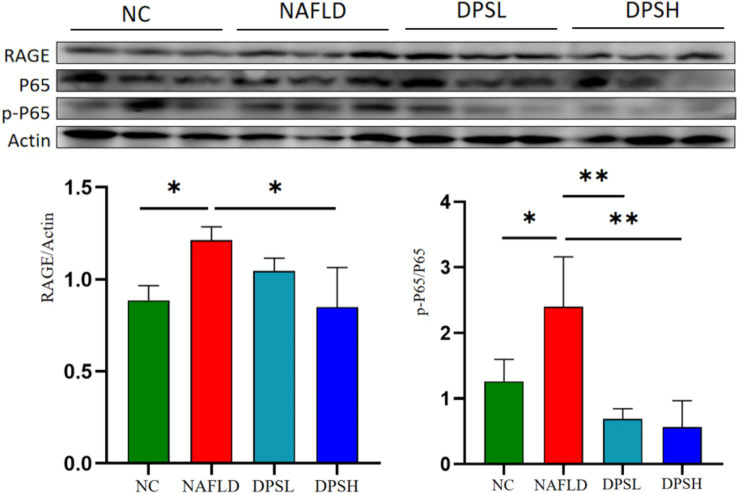
The expression of receptor for advanced glycation end products (RAGE) and the phosphorylation level of NF-κB P65 protein in mouse liver were detected using the Western blotting assay. *n* = 3. *: *p* < 0.05; **: *p* < 0.01.

RAGE, a receptor for advanced glycation end products (AGEs) and a novel pattern recognition receptor, plays a pivotal role in the pathogenesis of diseases such as diabetes, Alzheimer’s, and cancer. Western blotting results showed that the high-dose DPS (DPSH group) significantly reduced RAGE expression and NF-κB p65 protein phosphorylation in the liver of NAFLD mice (*p* < 0.05) (Figure 5).

### 3.5 DPS improves gut microbiota dysbiosis in NAFLD mice

**Figure 6:**
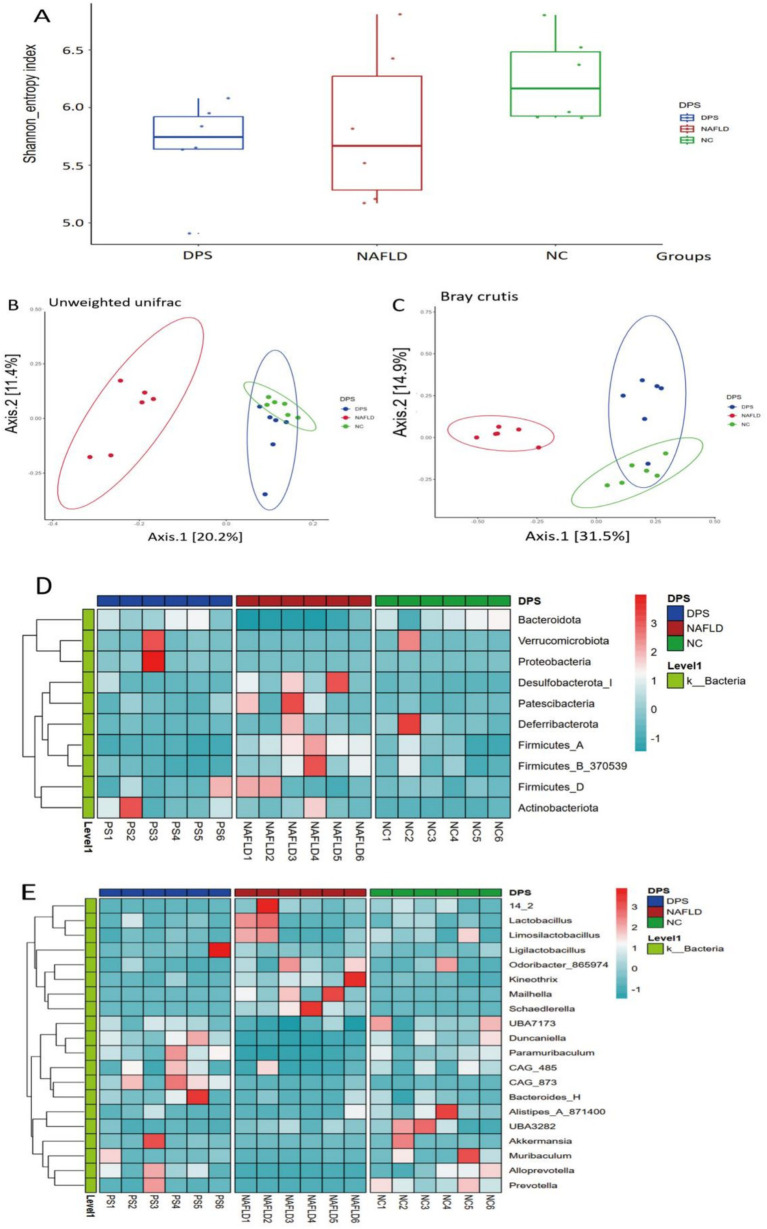
The gut microbiota of mice was analyzed using 16S rRNA amplicon sequencing. The α-diversity of shannon index **(A)**, β-diversity with unweighted unifrac **(B)** and bray curtis **(C)** of the gut microbiota in mice are shown, respectively. **(D,E)** Displayed the differences in the gut microbiota at the species level and genus level among the three groups of mice using heat maps. *n* = 6.

A high dose of DPS significantly inhibited liver inflammation and oxidative stress in NAFLD mice. This study further investigates the underlying mechanism of high-dose DPS treatment for NAFLD using gut microbiota analysis (*via* 16S rRNA sequencing) and serum non-targeted metabolomics. *α*-diversity analysis (Shannon index) showed no significant differences in microbial diversity among the three groups of mice (Figure 6A). *β*-diversity analysis (PCoA scatter plot) revealed distinct separation and clustering of the three groups, with DPS samples more closely resembling the NC group than the NAFLD group, suggesting that DPS ameliorates gut microbiota dysbiosis in NAFLD mice (Figure 6B).

Further analysis at the phylum and genus levels assessed the effects of oral DPS on the gut microbiota in NAFLD mice. At the phylum level, the NAFLD group exhibited a significant increase in the abundance of Patescibacteria, Firmicutes, and Actinobacteria, coupled with a notable decrease in Bacteroidetes, Verrucomicrobia, and Deferribacteres compared to the NC group (*p* < 0.05). Following DPS treatment, the DPS group showed a marked reduction in Patescibacteria, Deferribacteres, Firmicutes, and Actinobacteria, alongside a significant increase in Bacteroidetes, Verrucomicrobia, and Proteobacteria, relative to the NAFLD group (*p* < 0.05) (Figure 6C).

At the genus level, the NAFLD group exhibited a significant increase in the abundance of *Odoribacter* and *Mailhella*, while *Ligilactobacillus*, *Akkermansia*, and *Alistipes* were significantly reduced compared to the NC group (*p* < 0.05). After DPS intervention, the DPS group showed a significant decrease in *Odoribacter* and *Mailhella*, with a significant increase in the abundance of *Ligilactobacillus*, *Duncaniella*, and *Akkermansia* compared to the NAFLD group (*p* < 0.05) (Figure 6D).

### 3.6 DPS significantly improved serum metabolomics disorders in NAFLD mice

**Figure 7:**
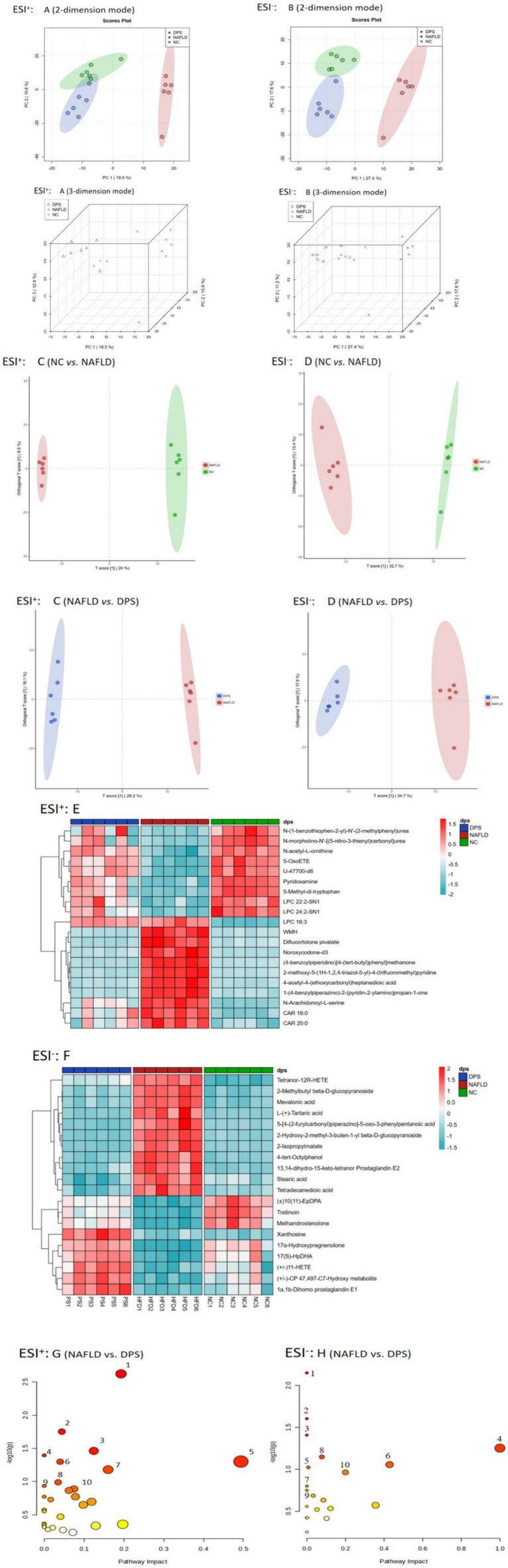
Untargeted metabolomics analysis of mouse serum. The PCA plot showed that samples from each group were significantly separated and clustered individually **(A,B)**. Through OPLS-DA model analysis **(C,D)**, differential metabolites were identified based on the screening criteria of VIP > 1 & *p* < 0.05 **(E,F)**. These metabolites were then input into the MetaboAnalyst 6.0 platform for enrichment analysis to obtain metabolic pathways **(G,H)**. *n* = 6.

The PCA plot revealed distinct differences in serum metabolomics among the three groups of mice. In both ESI + and ESI- modes, the NAFLD group samples formed distinct clusters, significantly separated from those of the NC and DPS groups. The DPS group samples closely resembled those of the NC group, indicating a significant restorative effect of DPS on serum metabolomic alterations in NAFLD mice (Figures 7A,B).

To identify differential metabolites, the OPLS-DA model was applied with criteria of VIP > 1 and *p* < 0.05 (Figures 7C,D). In the ESI + mode, 301 differential metabolites were identified between the NAFLD and DPS groups. Of these, 174 were highly expressed, and 127 were downregulated in the DPS group compared to the NAFLD group. In the ESI- mode, 255 differential metabolites were identified, with 93 highly expressed and 162 downregulated (Figures 7E,F). Metabolic pathway enrichment analysis, performed using the MetaboAnalyst 6.0 platform, revealed involvement in pathways such as arginine biosynthesis, glutathione metabolism, arginine and proline metabolism, unsaturated fatty acid biosynthesis, propionate metabolism, alanine, aspartate and glutamate metabolism, and linoleic acid metabolism (Figures 7G,H).

### 3.7 Relationship between gut microbiota and serum metabolites

**Figure 8:**
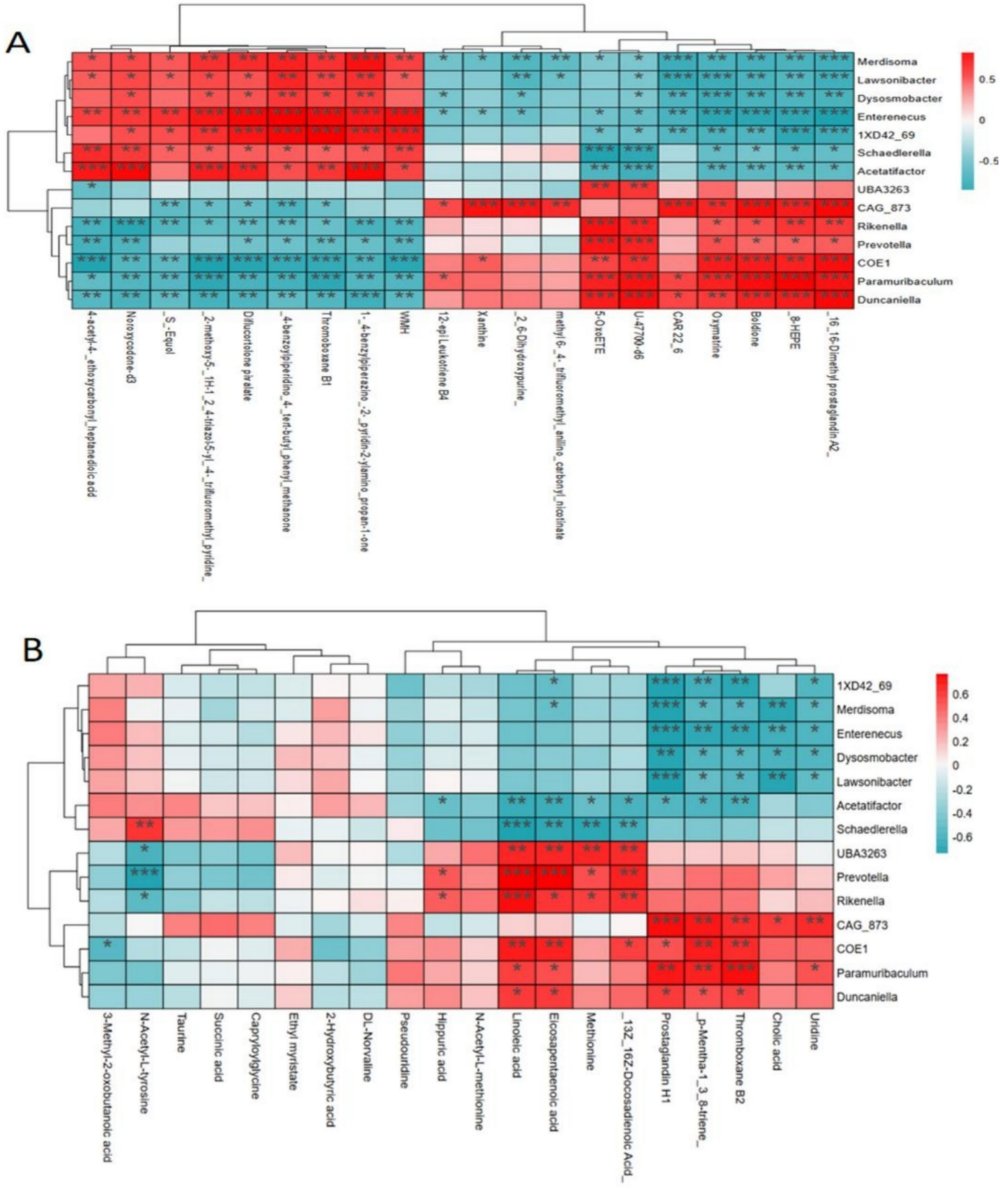
Pearson correlation analysis of the relationship between gut microbiota and serum metabolites. **(A)** ESI^+^ mode; **(B)** ESI^−^ mode. *n* = 6.

Pearson correlation analysis was conducted to explore the relationship between gut microbiota and serum metabolites in mice following DPS treatment, to further investigate the underlying mechanism of DPS in NAFLD. The results showed that serum levels of unsaturated fatty acids, specifically linoleic acid and eicosapentaenoic acid, were positively correlated with gut microbiota species such as *UBA3263*, *Prevotella*, *Rikenella*, and *COE1* while showing a negative correlation with *Schaedlerella* and *Acetatifactor*. Additionally, serum uridine levels were positively associated with gut microbiota *CAG_873* and *Paramuribaculum* but negatively correlated with *Merdisoma*, *Enterenecus*, *Dysosmobacter*, and *Lawsonibacter* (Figure 8).

## 4 Discussion

NAFLD, a metabolic disorder characterized by excessive hepatic fat accumulation, is a major contributor to liver fibrosis and cancer (29–31). The accumulation of lipids in the liver plays a pivotal role in disease progression through oxidative stress and inflammation (32–34). Furthermore, chronic inflammation resulting from endotoxins released into the bloodstream, due to the excessive proliferation of Gram-negative bacteria in the intestines, is a key factor in NAFLD progression (35, 36). Endotoxin levels in the blood can also serve as an indicator of NAFLD severity (37–39). The supplementation of anti-inflammatory and antioxidant agents, including *α*-lipoic acid, vitamin E, and various plant-derived antioxidants, represents an effective therapeutic approach for treating NAFLD (40–42).

Network pharmacology analysis identified the novel pattern recognition receptor RAGE and the inflammation-related NF-κB signaling pathway as key players in NAFLD pathogenesis. Initially, RAGE was regarded primarily as a receptor for AGEs (43, 44), which are formed through the non-enzymatic glycation of free sugars such as glucose and galactose (45, 46). Elevated blood glucose in diabetic patients leads to a marked increase in AGEs, which are pivotal in the development of diabetic complications (44, 47, 48). Recent studies have expanded the role of RAGE as a novel pattern recognition receptor, capable of binding not only with AGEs but also with ligands such as HMGB1, S100, and Aβ (49–52). RAGE’s functions are implicated in diseases related to homeostasis, development, and inflammation, including diabetes, atherosclerosis, and Alzheimer’s disease (49, 53, 54). Moreover, RAGE signaling in tumor and immune cells can drive tumor progression, migration, and immune evasion, promoting cancer development (55–58).

DPS is a rare sugar found in fruits such as figs and raisins, and it has been approved as a safe food additive by the U.S. FDA and the European Union (59). Acute/subchronic toxicity tests show that even with high doses (4 g/kg body weight/day) consumed over a long period (90 days), no significant organ damage or blood biochemical abnormalities were observed (60). Additionally, only about 30% of DPS is absorbed in the intestines, with the rest being fermented by intestinal flora (61). Its low-calorie characteristic (0.4 kcal/g) helps prevent exacerbation of NAFLD due to excess calorie intake (62). Compared to other sugar alcohols like erythritol, DPS has better gastrointestinal tolerance, and even daily intake of high doses of DPS (≤30 g/day) does not cause noticeable bloating or diarrhea, making it an ideal sugar substitute for individuals with obesity and diabetes (7).

Our study reveals that DPS significantly improves serum lipid profiles in HFD-induced NAFLD mice. These findings suggest that DPS exerts systemic metabolic benefits beyond its direct hepatic effects, potentially through multi-target modulation of cholesterol homeostasis. The AGEs/RAGE/NF-κB axis inhibition by DPS may restore hepatic LDL receptor (LDLR) functionality. However, the mechanism of DPS regulation of blood lipid metabolism still needs to be further studied.

In NAFLD, excessive lipid peroxides in the liver not only induce inflammation but also foster the formation of AGEs (63). Upon binding to RAGE, AGEs initiate intracellular signaling that generates reactive oxygen species, which can damage hepatocytes (44, 64–66). Recent studies suggest that activation of the AGEs/RAGE/NF-κB pathway plays a significant role in the complications of type 2 diabetes (50, 67–69). Inhibiting the RAGE and NF-κB signaling pathways may provide therapeutic benefits in preventing and treating diabetes complications (67, 70, 71).

Molecular docking analysis demonstrated that DPS effectively binds to both NF-κB p65 and RAGE proteins, significantly inhibiting the activation of both the NF-κB and AGEs/RAGE pathways. Western blot assays confirmed that DPS significantly reduces RAGE expression and NF-κB p65 protein phosphorylation in the livers of NAFLD mice. These findings suggest that DPS may alleviate inflammation and liver damage in NAFLD by modulating the AGEs/RAGE/NF-κB pathway.

Notably, our data reveal that DPS administration significantly attenuated hepatic MDA accumulation while enhancing SOD activity, suggesting its potent capacity to counteract the redox imbalance characteristic of NAFLD progression. Mounting evidence implicates AGEs as critical mediators in NAFLD pathogenesis, where their interaction with RAGE not only perpetuates inflammatory cascades *via* NF-κB activation but also directly amplifies oxidative damage through NADPH oxidase-driven ROS generation (72, 73). Our findings align with these mechanisms, as DPS treatment effectively suppressed RAGE overexpression and downstream NF-κB phosphorylation. This dual modulation likely disrupts the self-reinforcing cycle between AGEs accumulation and oxidative stress – a phenomenon particularly relevant in lipid-laden hepatocytes where *β*-oxidation overload exacerbates mitochondrial ROS production.

Furthermore, the antioxidant effects of DPS may synergize with its anti-inflammatory actions. NF-κB activation stimulates pro-oxidant enzymes while suppressing antioxidant genes, creating a pathogenic feedback loop. DPS-mediated NF-κB inhibition could therefore break this cycle. Such coordinated modulation of oxidative-inflammatory crosstalk positions DPS as a promising multi-target agent for NAFLD management.

Han et al. (74) demonstrated that DPS modulates gut microbiota and promotes the production of beneficial metabolites, such as short-chain fatty acids (SCFAs), while also alleviating diabetes and obesity in experimental animals (75). Through 16S rRNA sequencing, oral DPS administration was found to notably increase the abundance of beneficial gut bacteria, including *Akkermansia* and *Duncaniella*, in NAFLD mice, which are associated with SCFA production (76, 77). Furthermore, DPS enhanced the abundance of *Ligilactobacillus*, which plays a role in inhibiting liver fat accumulation and hyperlipidemia (78, 79). Significant improvements were also observed in oxidative stress and blood lipid levels in NAFLD mice. These findings suggest that DPS exerts therapeutic effects on NAFLD by reshaping gut microbiota and elevating beneficial metabolites like SCFAs.

Addressing gut dysbiosis represents an innovative approach to NAFLD management (80, 81). Both *Akkermansia* and *Duncaniella* contribute to NAFLD modulation by influencing gut and liver functions. *Akkermansia* reduces TLR2 expression and macrophage activation (82), while *Duncaniella* regulates the production of 3,7-dihydroxy-12-oxocholanoic acid, inhibiting hepatic gluconeogenesis and lipid metabolism (83).

Untargeted metabolomics analysis further revealed that DPS significantly alters the serum metabolome in NAFLD mice, boosting the levels of anti-inflammatory and antioxidant metabolites. Notably, tretinoin, a bioactive metabolite of vitamin A, is found at markedly lower concentrations in the serum of patients with NAFLD compared to healthy controls (84). Tretinoin promotes the reduction of fat deposition and ameliorates NAFLD symptoms by enhancing fatty acid *β*-oxidation in the liver (85). Additionally, it may enhance liver antioxidant capacity *via* the Sirt1 pathway, thereby mitigating high-fat diet-induced liver steatosis (86). These findings suggest that DPS treatment elevates serum tretinoin levels in NAFLD mice, providing a potential mechanism for its therapeutic action in NAFLD. Pyridoxamine, a derivative of vitamin B6, is critical for preventing multiple diseases when deficient, although its role in metabolic syndrome remains underexplored. Patients with NAFLD exhibit significantly lower serum pyridoxamine levels than healthy individuals (87). Pyridoxamine prevents AGE formation (88), improves lipid metabolism in NAFLD rats (89), and reduces hepatic lipid peroxidation and inflammation (90). Our data indicate that oral DPS administration significantly increases serum pyridoxamine concentrations in NAFLD mice, suggesting that this elevation may suppress AGEs-RAGE pathway activation, thereby mitigating inflammation and oxidative stress in NAFLD.

Statins regulate blood lipids and exert anti-inflammatory effects by inhibiting mevalonic acid synthesis in the liver and activating hepatic stellate cells, thereby preventing the progression of liver fibrosis (91–93). In this study, DPS also significantly reduced serum mevalonic acid levels in NAFLD mice, suggesting that DPS may regulate blood lipids and mitigate inflammatory liver damage *via* the mevalonic acid synthesis pathway.

The study further identified increased levels of eicosapentaenoic acid (EPA) and linoleic acid in DPS-treated mice, with a significant positive correlation between these unsaturated fatty acids and the relative abundance of *Duncaniella*. Clinical studies have shown that elevated EPA concentrations in the serum of patients with cirrhosis are associated with a reduced risk of progression to liver cancer (94). EPA exhibits potent anti-inflammatory and antioxidant properties, which can significantly prevent liver cell degeneration and fibrosis in patients with NAFLD (95). Linoleic acid, a gut microbial metabolite, inhibits the activation of the TGF-*β* signaling pathway in hepatic stellate cells, thus preventing liver fibrosis progression (18). These findings suggest that DPS significantly increases serum linoleic acid levels in NAFLD mice, pointing to a potential mechanism underlying DPS’s therapeutic effects on NAFLD.

## 5 Conclusion

Our results indicate that oral DPS administration effectively regulates blood lipids in NAFLD mice and ameliorates inflammation and oxidative stress in the liver. The therapeutic action of DPS in NAFLD likely involves the modulation of gut microbiota and the enhancement of anti-inflammatory and antioxidant metabolites in the serum.

However, we believe that the biggest obstacle for DPS from the laboratory to the market is its high production cost, which is more than 2–3 times the cost of alternative sweeteners such as erythritol and steviol glycosides. We think that DPS therapy might be more suitable for prioritization in high-risk populations for NAFLD (such as pre-diabetic patients) rather than the general healthy population, in order to reduce medical costs. This is because the cost of using DPS therapy to prevent the progression of NAFLD is lower compared to the treatment expenses after NAFLD progresses to NASH.

## Data Availability

The original contributions presented in the study are publicly available. This data can be found here: https://ngdc.cncb.ac.cn/search/all?&q=PRJCA039581.

## Footnotes

^1^ http://omim.org/

^2^ https://www.genecards.org/

^3^ https://www.rstudio.com/

^4^ https://david.ncifcrf.gov/

^5^ http://www.resb.org/

^6^ https://bioincloud.tech/

## References

[1] Jung YS Kim HG Cho CH Lee SH Lee N Yang J. Trapping mechanism by di-d-psicose anhydride with methylglyoxal for prevention of diabetic nephropathy. Carbohydr Res. (2024) 540:109125. doi: 10.1016/j.carres.2024.109125, PMID: 38703663

[2] Song Y Maskey S Lee YG Lee DS Nguyen DT Bae HJ. Optimizing bioconversion processes of rice husk into value-added products: D-psicose, bioethanol, and lactic acid. Bioresour Technol. (2024) 395:130363. doi: 10.1016/j.biortech.2024.130363 38253244

[3] Natsume Y Yamada T Iida T Ozaki N Gou Y Oshida Y. Investigation of d-allulose effects on high-sucrose diet-induced insulin resistance via hyperinsulinemic-euglycemic clamps in rats. Heliyon. (2021) 7:e08013. doi: 10.1016/j.heliyon.2021.e08013, PMID: 34589631 PMC8461346

[4] Teysseire F Bordier V Budzinska A Weltens N Rehfeld JF Holst JJ. The role of D-allulose and erythritol on the activity of the gut sweet taste receptor and gastrointestinal satiation hormone release in humans: a randomized, controlled trial. J Nutr. (2022) 152:1228–38. doi: 10.1093/jn/nxac026, PMID: 35135006 PMC9071322

[5] Watthanasakphuban N Srila P Pinmanee P Punvittayagul C Petchyam N Ninchan B. Production, purification, characterization, and safety evaluation of constructed recombinant D-psicose 3-epimerase. Microb Cell Factories. (2024) 23:216. doi: 10.1186/s12934-024-02487-x, PMID: 39080612 PMC11290309

[6] Van Laar A Grootaert C Van Camp J. Rare mono- and disaccharides as healthy alternative for traditional sugars and sweeteners. Crit Rev Food Sci Nutr. (2021) 61:713–41. doi: 10.1080/10408398.2020.1743966, PMID: 32212974

[7] Lei P Chen H Ma J Fang Y Qu L Yang Q. Research progress on extraction technology and biomedical function of natural sugar substitutes. Front Nutr. (2022) 9:952147. doi: 10.3389/fnut.2022.952147, PMID: 36034890 PMC9414081

[8] Li J Li H Liu H Luo Y. Recent advances in the biosynthesis of natural sugar substitutes in yeast. J Fungi (Basel). (2023) 9:907. doi: 10.3390/jof9090907, PMID: 37755015 PMC10533046

[9] Kanasaki A Niibo M Iida T. Effect of D-allulose feeding on the hepatic metabolomics profile in male Wistar rats. Food Funct. (2021) 12:3931–8. doi: 10.1039/d0fo03024d, PMID: 33977954

[10] Xie X Li C Ban X Yang H Li Z. D-allulose 3-epimerase for low-calorie D-allulose synthesis: microbial production, characterization, and applications. Crit Rev Biotechnol. (2024) 45:1–20. doi: 10.1080/07388551.2024.2368517 38973014

[11] Mantovani A Csermely A Petracca G Beatrice G Corey KE Simon TG. Non-alcoholic fatty liver disease and risk of fatal and non-fatal cardiovascular events: an updated systematic review and meta-analysis. Lancet Gastroenterol Hepatol. (2021) 6:903–13. doi: 10.1016/S2468-1253(21)00308-3, PMID: 34555346

[12] Quek J Chan KE Wong ZY Tan C Tan B Lim WH. Global prevalence of non-alcoholic fatty liver disease and non-alcoholic steatohepatitis in the overweight and obese population: a systematic review and meta-analysis. Lancet Gastroenterol Hepatol. (2023) 8:20–30. doi: 10.1016/S2468-1253(22)00317-X, PMID: 36400097

[13] Tacke F Weiskirchen R. Non-alcoholic fatty liver disease (NAFLD)/non-alcoholic steatohepatitis (NASH)-related liver fibrosis: mechanisms, treatment and prevention. Ann Transl Med. (2021) 9:729. doi: 10.21037/atm-20-4354, PMID: 33987427 PMC8106094

[14] Ramai D Facciorusso A Vigandt E Schaf B Saadedeen W Chauhan A. Progressive liver fibrosis in non-alcoholic fatty liver disease. Cells. (2021) 10:3401. doi: 10.3390/cells10123401, PMID: 34943908 PMC8699709

[15] Gallage S Avila J Ramadori P Focaccia E Rahbari M Ali A. A researcher's guide to preclinical mouse NASH models. Nat Metab. (2022) 4:1632–49. doi: 10.1038/s42255-022-00700-y, PMID: 36539621

[16] Muzurović E Mikhailidis DP Mantzoros C. Non-alcoholic fatty liver disease, insulin resistance, metabolic syndrome and their association with vascular risk. Metabolism. (2021) 119:154770. doi: 10.1016/j.metabol.2021.154770 33864798

[17] Ziolkowska S Binienda A Jabłkowski M Szemraj J Czarny P. The interplay between insulin resistance, inflammation, oxidative stress, base excision repair and metabolic syndrome in nonalcoholic fatty liver disease. Int J Mol Sci. (2021) 22:11128. doi: 10.3390/ijms222011128, PMID: 34681787 PMC8537238

[18] Kasahara N Imi Y Amano R Shinohara M Okada K Hosokawa Y. A gut microbial metabolite of linoleic acid ameliorates liver fibrosis by inhibiting TGF-β signaling in hepatic stellate cells. Sci Rep. (2023) 13:18983. doi: 10.1038/s41598-023-46404-5, PMID: 37923895 PMC10624680

[19] Attia SL Softic S Mouzaki M. Evolving role for pharmacotherapy in NAFLD/NASH. Clin Transl Sci. (2021) 14:11–9. doi: 10.1111/cts.12839, PMID: 32583961 PMC7877845

[20] Pafili K Roden M. Nonalcoholic fatty liver disease (NAFLD) from pathogenesis to treatment concepts in humans. Mol Metab. (2021) 50:101122. doi: 10.1016/j.molmet.2020.101122, PMID: 33220492 PMC8324683

[21] Lee YA Friedman SL. Inflammatory and fibrotic mechanisms in NAFLD-implications for new treatment strategies. J Intern Med. (2022) 291:11–31. doi: 10.1111/joim.13380, PMID: 34564899 PMC8688191

[22] Kokkorakis M Boutari C Hill MA Kotsis V Loomba R Sanyal AJ. Resmetirom, the first approved drug for the management of metabolic dysfunction-associated steatohepatitis: trials, opportunities, and challenges. Metabolism. (2024) 154:155835. doi: 10.1016/j.metabol.2024.155835 38508373

[23] Chen VL Morgan TR Rotman Y Patton HM Cusi K Kanwal F. Resmetirom therapy for metabolic dysfunction-associated steatotic liver disease: October 2024 updates to AASLD practice guidance. Hepatology. (2025) 81:312–20. doi: 10.1097/HEP.0000000000001112 39422487

[24] Raza S Rajak S Upadhyay A Tewari A Anthony SR. Current treatment paradigms and emerging therapies for NAFLD/NASH. Front Biosci (Landmark Ed). (2021) 26:206–37. doi: 10.2741/4892 33049668 PMC7116261

[25] Kobayashi T Iwaki M Nakajima A Nogami A Yoneda M. Current research on the pathogenesis of NAFLD/NASH and the gut-liver axis: gut microbiota, dysbiosis, and leaky-gut syndrome. Int J Mol Sci. (2022) 23:11689. doi: 10.3390/ijms231911689, PMID: 36232990 PMC9570241

[26] Vetrano E Rinaldi L Mormone A Giorgione C Galiero R Caturano A. Non-alcoholic fatty liver disease (NAFLD), type 2 diabetes, and non-viral hepatocarcinoma: pathophysiological mechanisms and new therapeutic strategies. Biomedicines. (2023) 11:468. doi: 10.3390/biomedicines11020468, PMID: 36831004 PMC9953066

[27] Sun C Qiu C Zhang Y Yan M Tan J He J. Lactiplantibacillus plantarum NKK20 alleviates high-fat-diet-induced nonalcoholic fatty liver disease in mice through regulating bile acid anabolism. Molecules. (2023) 28:4042. doi: 10.3390/molecules28104042, PMID: 37241783 PMC10222591

[28] He J Li X Yan M Chen X Sun C Tan J. Inulin reduces kidney damage in type 2 diabetic mice by decreasing inflammation and serum metabolomics. J Diabetes Res. (2024) 2024:1222395. doi: 10.1155/2024/1222395, PMID: 38725443 PMC11081752

[29] Geng Y Faber KN de Meijer VE Blokzijl H Moshage H. How does hepatic lipid accumulation lead to lipotoxicity in non-alcoholic fatty liver disease. Hepatol Int. (2021) 15:21–35. doi: 10.1007/s12072-020-10121-2, PMID: 33548031 PMC7886759

[30] Fujiwara N Kubota N Crouchet E Koneru B Marquez CA Jajoriya AK. Molecular signatures of long-term hepatocellular carcinoma risk in nonalcoholic fatty liver disease. Sci Transl Med. (2022) 14:eabo4474. doi: 10.1126/scitranslmed.abo4474, PMID: 35731891 PMC9236162

[31] Guo W Ge X Lu J Xu X Gao J Wang Q. Diet and risk of non-alcoholic fatty liver disease, cirrhosis, and liver cancer: a large prospective cohort study in UK biobank. Nutrients. (2022) 14:5335. doi: 10.3390/nu14245335, PMID: 36558494 PMC9788291

[32] Madduma Hewage S Prashar S Karmin O Siow YL. Lingonberry improves non-alcoholic fatty liver disease by reducing hepatic lipid accumulation, oxidative stress and inflammatory response. Antioxidants (Basel). (2021) 10:565. doi: 10.3390/antiox10040565, PMID: 33917360 PMC8067338

[33] Martín-Fernández M Arroyo V Carnicero C Sigüenza R Busta R Mora N. Role of oxidative stress and lipid peroxidation in the pathophysiology of NAFLD. Antioxidants (Basel). (2022) 11:2217. doi: 10.3390/antiox11112217, PMID: 36358589 PMC9686676

[34] Li L Wang YM Zeng XY Hu Y Zhang J Wang B. Bioactive proteins and antioxidant peptides from *Litsea cubeba* fruit meal: preparation, characterization and ameliorating function on high-fat diet-induced NAFLD through regulating lipid metabolism, oxidative stress and inflammatory response. Int J Biol Macromol. (2024) 280:136186. doi: 10.1016/j.ijbiomac.2024.136186, PMID: 39357720

[35] Kessoku T Kobayashi T Imajo K Tanaka K Yamamoto A Takahashi K. Endotoxins and non-alcoholic fatty liver disease. Front Endocrinol (Lausanne). (2021) 12:770986. doi: 10.3389/fendo.2021.770986, PMID: 34777261 PMC8586459

[36] Bergheim I Moreno-Navarrete JM. The relevance of intestinal barrier dysfunction, antimicrobial proteins and bacterial endotoxin in metabolic dysfunction-associated steatotic liver disease. Eur J Clin Investig. (2024) 54:e14224. doi: 10.1111/eci.14224 38634717

[37] Barchetta I Cimini FA Sentinelli F Chiappetta C Di Cristofano C Silecchia G. Reduced lipopolysaccharide-binding protein (LBP) levels are associated with non-alcoholic fatty liver disease (NAFLD) and adipose inflammation in human obesity. Int J Mol Sci. (2023) 24:17174. doi: 10.3390/ijms242417174, PMID: 38139003 PMC10742626

[38] Soppert J Brandt EF Heussen NM Barzakova E Blank LM Kuepfer L. Blood endotoxin levels as biomarker of nonalcoholic fatty liver disease: a systematic review and meta-analysis. Clin Gastroenterol Hepatol. (2023) 21:2746–58. doi: 10.1016/j.cgh.2022.11.030, PMID: 36470528

[39] Yuan H Wu X Wang X Zhou JY Park S. Microbial dysbiosis linked to metabolic dysfunction-associated fatty liver disease in Asians: *Prevotella copri* promotes lipopolysaccharide biosynthesis and network instability in the prevotella enterotype. Int J Mol Sci. (2024) 25:2183. doi: 10.3390/ijms25042183, PMID: 38396863 PMC10889285

[40] Mosca A Crudele A Smeriglio A Braghini MR Panera N Comparcola D. Antioxidant activity of hydroxytyrosol and vitamin E reduces systemic inflammation in children with paediatric NAFLD. Dig Liver Dis. (2021) 53:1154–8. doi: 10.1016/j.dld.2020.09.021 33060043

[41] Tutunchi H Zolrahim F Nikbaf-Shandiz M Naeini F Ostadrahimi A Naghshi S. Effects of oleoylethanolamide supplementation on inflammatory biomarkers, oxidative stress and antioxidant parameters of obese patients with NAFLD on a calorie-restricted diet: a randomized controlled trial. Front Pharmacol. (2023) 14:1144550. doi: 10.3389/fphar.2023.1144550, PMID: 37089938 PMC10119414

[42] Mohammadian K Fakhar F Keramat S Stanek A. The role of antioxidants in the treatment of metabolic dysfunction-associated fatty liver disease: a systematic review. Antioxidants (Basel). (2024) 13:797. doi: 10.3390/antiox13070797, PMID: 39061866 PMC11273623

[43] Twarda-Clapa A Olczak A Białkowska AM Koziołkiewicz M. Advanced glycation end-products (AGEs): formation, chemistry, classification, receptors, and diseases related to AGEs. Cells. (2022) 11:1312. doi: 10.3390/cells11081312, PMID: 35455991 PMC9029922

[44] Wu XQ Zhang DD Wang YN Tan YQ Yu XY Zhao YY. AGE/RAGE in diabetic kidney disease and ageing kidney. Free Radic Biol Med. (2021) 171:260–71. doi: 10.1016/j.freeradbiomed.2021.05.025 34019934

[45] Wu Q Liang Y Kong Y Zhang F Feng Y Ouyang Y. Role of glycated proteins in vivo: enzymatic glycated proteins and non-enzymatic glycated proteins. Food Res Int. (2022) 155:111099. doi: 10.1016/j.foodres.2022.111099, PMID: 35400472

[46] Shen CY Lu CH Cheng CF Li KJ Kuo YM Wu CH. Advanced glycation end-products acting as immunomodulators for chronic inflammation, inflammaging and carcinogenesis in patients with diabetes and immune-related diseases. Biomedicines. (2024) 12:1699. doi: 10.3390/biomedicines12081699, PMID: 39200164 PMC11352041

[47] Deng L Du C Song P Chen T Rui S Armstrong DG. The role of oxidative stress and antioxidants in diabetic wound healing. Oxidative Med Cell Longev. (2021) 2021:8852759. doi: 10.1155/2021/8852759, PMID: 33628388 PMC7884160

[48] Samsu N. Diabetic nephropathy: challenges in pathogenesis, diagnosis, and treatment. Biomed Res Int. (2021) 2021:1497449. doi: 10.1155/2021/1497449, PMID: 34307650 PMC8285185

[49] Dong H Zhang Y Huang Y Deng H. Pathophysiology of RAGE in inflammatory diseases. Front Immunol. (2022) 13:931473. doi: 10.3389/fimmu.2022.931473, PMID: 35967420 PMC9373849

[50] Liu S Zhang Y Yang F Gu J Zhang R Kuang Y. Modified Cangfu Daotan decoction ameliorates polycystic ovary syndrome with insulin resistance via NF-κB/LCN-2 signaling pathway in inflammatory microenvironment. Front Endocrinol (Lausanne). (2022) 13:975724. doi: 10.3389/fendo.2022.975724, PMID: 36440213 PMC9686851

[51] Maheshwari S. AGEs RAGE pathways: Alzheimer's disease. Drug Res (Stuttg). (2023) 73:251–4. doi: 10.1055/a-2008-7948, PMID: 36940723

[52] Sarkar S. Pathological role of RAGE underlying progression of various diseases: its potential as biomarker and therapeutic target. Naunyn Schmiedeberg's Arch Pharmacol. (2024) 398:3467–87. doi: 10.1007/s00210-024-03595-6, PMID: 39589529

[53] Juranek J Mukherjee K Kordas B Załęcki M Korytko A Zglejc-Waszak K. Role of RAGE in the pathogenesis of neurological disorders. Neurosci Bull. (2022) 38:1248–62. doi: 10.1007/s12264-022-00878-x, PMID: 35729453 PMC9554177

[54] Zhou M Zhang Y Shi L Li L Zhang D Gong Z. Activation and modulation of the AGEs-RAGE axis: implications for inflammatory pathologies and therapeutic interventions – a review. Pharmacol Res. (2024) 206:107282. doi: 10.1016/j.phrs.2024.107282, PMID: 38914383

[55] Taneja S Vetter SW Leclerc E. Hypoxia and the receptor for advanced glycation end products (RAGE) signaling in cancer. Int J Mol Sci. (2021) 22:8153. doi: 10.3390/ijms22158153 34360919 PMC8348933

[56] Waghela BN Vaidya FU Ranjan K Chhipa AS Tiwari BS Pathak C. AGE-RAGE synergy influences programmed cell death signaling to promote cancer. Mol Cell Biochem. (2021) 476:585–98. doi: 10.1007/s11010-020-03928-y, PMID: 33025314

[57] Amornsupak K Thongchot S Thinyakul C Box C Hedayat S Thuwajit P. HMGB1 mediates invasion and PD-L1 expression through RAGE-PI3K/AKT signaling pathway in MDA-MB-231 breast cancer cells. BMC Cancer. (2022) 22:578. doi: 10.1186/s12885-022-09675-1, PMID: 35610613 PMC9128129

[58] Park WY Gray JM Holewinski RJ Andresson T So JY Carmona-Rivera C. Apoptosis-induced nuclear expulsion in tumor cells drives S100a4-mediated metastatic outgrowth through the RAGE pathway. Nat Cancer. (2023) 4:419–35. doi: 10.1038/s43018-023-00524-z, PMID: 36973439 PMC10042736

[59] Chen J Chen D Ke M Ye S Wang X Zhang W. Characterization of a recombinant D-Allulose 3-epimerase from Thermoclostridium caenicola with potential application in D-Allulose production. Mol Biotechnol. (2021) 63:534–43. doi: 10.1007/s12033-021-00320-z, PMID: 33782841

[60] Matsuo T Ishii R Shirai Y. The 90-day oral toxicity of d-psicose in male Wistar rats. J Clin Biochem Nutr. (2012) 50:158–61. doi: 10.3164/jcbn.11-66, PMID: 22448098 PMC3303479

[61] Li J Dai Q Zhu Y Xu W Zhang W Chen Y. Low-calorie bulk sweeteners: recent advances in physical benefits, applications, and bioproduction. Crit Rev Food Sci Nutr. (2024) 64:6581–95. doi: 10.1080/10408398.2023.2171362, PMID: 36705477

[62] Iida T Hayashi N Yamada T Yoshikawa Y Miyazato S Kishimoto Y. Failure of d-psicose absorbed in the small intestine to metabolize into energy and its low large intestinal fermentability in humans. Metabolism. (2010) 59:206–14. doi: 10.1016/j.metabol.2009.07.018 19765780

[63] Pang Q Sun Z Shao C Cai H Bao Z Wang L. CML/RAGE signal bridges a common pathogenesis between atherosclerosis and non-alcoholic fatty liver. Front Med (Lausanne). (2020) 7:583943. doi: 10.3389/fmed.2020.583943, PMID: 33240906 PMC7677500

[64] Jahan H Choudhary MI. Gliclazide alters macrophages polarization state in diabetic atherosclerosis in vitro via blocking AGE-RAGE/TLR4-reactive oxygen species-activated NF-kβ nexus. Eur J Pharmacol. (2021) 894:173874. doi: 10.1016/j.ejphar.2021.173874, PMID: 33460615

[65] Abouelezz HM Shehatou G Shebl AM Salem HA. A standardized pomegranate fruit extract ameliorates thioacetamide-induced liver fibrosis in rats via AGE-RAGE-ROS signaling. Heliyon. (2023) 9:e14256. doi: 10.1016/j.heliyon.2023.e14256, PMID: 36938469 PMC10015255

[66] Sakasai-Sakai A Takeda K Takeuchi M. Involvement of intracellular TAGE and the TAGE-RAGE-ROS axis in the onset and progression of NAFLD/NASH. Antioxidants (Basel). (2023) 12:748. doi: 10.3390/antiox12030748, PMID: 36978995 PMC10045097

[67] Mazumder K Biswas B Al Mamun A Billah H Abid A Sarkar KK. Investigations of AGEs' inhibitory and nephroprotective potential of ursolic acid towards reduction of diabetic complications. J Nat Med. (2022) 76:490–503. doi: 10.1007/s11418-021-01602-1, PMID: 35032247

[68] Horvat A Vlašić I Štefulj J Oršolić N Jazvinšćak JM. Flavonols as a potential pharmacological intervention for alleviating cognitive decline in diabetes: evidence from preclinical studies. Life (Basel). (2023) 13:2291. doi: 10.3390/life13122291, PMID: 38137892 PMC10744738

[69] Apte MM Khattar E Tupe RS. Mechanistic role of *Syzygium cumini* (L.) Skeels in glycation induced diabetic nephropathy via RAGE-NF-κB pathway and extracellular proteins modifications: a molecular approach. J Ethnopharmacol. (2024) 322:117573. doi: 10.1016/j.jep.2023.117573, PMID: 38110133

[70] Nguyen HN Ullevig SL Short JD Wang L Ahn YJ Asmis R. Ursolic acid and related analogues: triterpenoids with broad health benefits. Antioxidants (Basel). (2021) 10:1161. doi: 10.3390/antiox10081161, PMID: 34439409 PMC8388988

[71] Zhuang LG Zhang R Jin GX Pei XY Wang Q Ge XX. Asiaticoside improves diabetic nephropathy by reducing inflammation, oxidative stress, and fibrosis: an in vitro and in vivo study. World J Diabetes. (2024) 15:2111–22. doi: 10.4239/wjd.v15.i10.2111, PMID: 39493557 PMC11525727

[72] Milione S Di Caterino M Monaco L Rinaldi L. Mediation of inflammation, obesity and fatty liver disease by advanced glycation endoproducts. Eur Rev Med Pharmacol Sci. (2018) 22:578–9. doi: 10.26355/eurrev_201802_14267 29461583

[73] Fernando DH Forbes JM Angus PW Herath CB. Development and progression of non-alcoholic fatty liver disease: the role of advanced glycation end products. Int J Mol Sci. (2019) 20:5037. doi: 10.3390/ijms20205037, PMID: 31614491 PMC6834322

[74] Han Y Park H Choi BR Ji Y Kwon EY Choi MS. Alteration of microbiome profile by D-allulose in amelioration of high-fat-diet-induced obesity in mice. Nutrients. (2020) 12:352. doi: 10.3390/nu12020352, PMID: 32013116 PMC7071329

[75] Baek SH Park SJ Lee HG. D-psicose, a sweet monosaccharide, ameliorate hyperglycemia, and dyslipidemia in C57BL/6J db/db mice. J Food Sci. (2010) 75:H49–53. doi: 10.1111/j.1750-3841.2009.01434.x, PMID: 20492234

[76] Feng P Li Q Liu L Wang S Wu Z Tao Y. Crocetin prolongs recovery period of DSS-induced colitis via altering intestinal microbiome and increasing intestinal permeability. Int J Mol Sci. (2022) 23:3832. doi: 10.3390/ijms23073832, PMID: 35409192 PMC8998954

[77] Zhu Y Chen B Zhang X Akbar MT Wu T Zhang Y. Exploration of the Muribaculaceae family in the gut microbiota: diversity, metabolism, and function. Nutrients. (2024) 16:2660. doi: 10.3390/nu16162660, PMID: 39203797 PMC11356848

[78] Liang C Zhou XH Jiao YH Guo MJ Meng L Gong PM. Ligilactobacillus Salivarius LCK11 prevents obesity by promoting PYY secretion to inhibit appetite and regulating gut microbiota in C57BL/6J mice. Mol Nutr Food Res. (2021) 65:e2100136. doi: 10.1002/mnfr.202100136, PMID: 34272917

[79] Wei B Peng Z Xiao M Huang T Yang S Liu K. Modulation of the microbiome-fat-liver axis by lactic acid bacteria: a potential alleviated role in high-fat-diet-induced obese mice. J Agric Food Chem. (2023) 71:10361–74. doi: 10.1021/acs.jafc.3c03149, PMID: 37390401

[80] Leung H Long X Ni Y Qian L Nychas E Siliceo SL. Risk assessment with gut microbiome and metabolite markers in NAFLD development. Sci Transl Med. (2022) 14:eabk0855. doi: 10.1126/scitranslmed.abk0855, PMID: 35675435 PMC9746350

[81] Kuang J Wang J Li Y Li M Zhao M Ge K. Hyodeoxycholic acid alleviates non-alcoholic fatty liver disease through modulating the gut-liver axis. Cell Metab. (2023) 35:1752–66.e8. doi: 10.1016/j.cmet.2023.07.011, PMID: 37591244

[82] Han Y Ling Q Wu L Wang X Wang Z Chen J. *Akkermansia muciniphila* inhibits nonalcoholic steatohepatitis by orchestrating TLR2-activated γδT17 cell and macrophage polarization. Gut Microbes. (2023) 15:2221485. doi: 10.1080/19490976.2023.2221485, PMID: 37345844 PMC10288935

[83] Lin J Zhang R Liu H Zhu Y Dong N Qu Q. Multi-omics analysis of the biological mechanism of the pathogenesis of non-alcoholic fatty liver disease. Front Microbiol. (2024) 15:1379064. doi: 10.3389/fmicb.2024.1379064, PMID: 39132138 PMC11310135

[84] Liu Y Chen H Wang J Zhou W Sun R Xia M. Association of serum retinoic acid with hepatic steatosis and liver injury in nonalcoholic fatty liver disease. Am J Clin Nutr. (2015) 102:130–7. doi: 10.3945/ajcn.114.105155, PMID: 25948673

[85] Zhu S Zhang J Zhu D Jiang X Wei L Wang W. Adipose tissue plays a major role in retinoic acid-mediated metabolic homoeostasis. Adipocytes. (2022) 11:47–55. doi: 10.1080/21623945.2021.2015864, PMID: 34957917 PMC8726720

[86] Geng C Xu H Zhang Y Gao Y Li M Liu X. Retinoic acid ameliorates high-fat diet-induced liver steatosis through sirt1. Sci China Life Sci. (2017) 60:1234–41. doi: 10.1007/s11427-016-9027-6, PMID: 28667519

[87] Liu Z Li P Zhao ZH Zhang Y Ma ZM Wang SX. Vitamin B6 prevents endothelial dysfunction, insulin resistance, and hepatic lipid accumulation in apoe (−/−) mice fed with high-fat diet. J Diabetes Res. (2016) 2016:1748065. doi: 10.1155/2016/1748065, PMID: 26881239 PMC4735993

[88] Reeve EH Kronquist EK Wolf JR Lee B Khurana A Pham H. Pyridoxamine treatment ameliorates large artery stiffening and cerebral artery endothelial dysfunction in old mice. J Cereb Blood Flow Metab. (2023) 43:281–95. doi: 10.1177/0271678X221130124, PMID: 36189840 PMC9903220

[89] Pereira E Silvares RR Rodrigues KL Flores E Daliry A. Pyridoxamine and caloric restriction improve metabolic and microcirculatory abnormalities in rats with non-alcoholic fatty liver disease. J Vasc Res. (2021) 58:1–10. doi: 10.1159/000512832, PMID: 33535220

[90] Pereira E Silvares RR Flores E Rodrigues KL Daliry A. Pyridoxamine improves metabolic and microcirculatory complications associated with nonalcoholic fatty liver disease. Microcirculation. (2020) 27:e12603. doi: 10.1111/micc.12603, PMID: 31876010

[91] Quist-Paulsen P. Statins and inflammation: an update. Curr Opin Cardiol. (2010) 25:399–405. doi: 10.1097/HCO.0b013e3283398e53 20421792

[92] Satny M Hubacek JA Vrablik M. Statins and inflammation. Curr Atheroscler Rep. (2021) 23:80. doi: 10.1007/s11883-021-00977-6 34851454

[93] Kitsugi K Noritake H Matsumoto M Hanaoka T Umemura M Yamashita M. Simvastatin inhibits hepatic stellate cells activation by regulating the ferroptosis signaling pathway. Biochim Biophys Acta Mol basis Dis. (2023) 1869:166750. doi: 10.1016/j.bbadis.2023.166750 37268254

[94] Khan IM Gjuka D Jiao J Song X Wang Y Wang J. A novel biomarker panel for the early detection and risk assessment of hepatocellular carcinoma in patients with cirrhosis. Cancer Prev Res (Phila). (2021) 14:667–74. doi: 10.1158/1940-6207.CAPR-20-0600, PMID: 33685927 PMC8225562

[95] Tanaka N Sano K Horiuchi A Tanaka E Kiyosawa K Aoyama T. Highly purified eicosapentaenoic acid treatment improves nonalcoholic steatohepatitis. J Clin Gastroenterol. (2008) 42:413–8. doi: 10.1097/MCG.0b013e31815591aa, PMID: 18277895

