# Supplementary material for: D-Psicose mitigates NAFLD mice induced by a high-fat diet by reducing lipid accumulation, inflammation, and oxidative stress
Source: Front Nutr. 2025 May 27;12:1574151. doi: 10.3389/fnut.2025.1574151 (PMC12148910; doi:10.3389/fnut.2025.1574151)
Supplement: Supplementary file 1 [file Table_1.docx]

Table S1. Feed energy supply of mice

| Components | Normal Diet (kcal/gm) | High-Fat Diet (kcal/gm) |
| --- | --- | --- |
| Casein | 800/200 | 800/200 |
| L-Cystine | 12/3 | 12/3 |
| Corn Starch | 2024.8/506.2 | 0/0 |
| Maltodextrin | 500/125 | 500/125 |
| Sucrose | 275/68.8 | 275/68.8 |
| Cellulose | 0/50 | 0/50 |
| Soybean Oil | 225/25 | 225/25 |
| Lard | 180/20 | 2205/245 |
| Mineral Mix | 0/10 | 0/10 |
| DiCalcium Phosphate | 0/13 | 0/13 |
| Calcium Carbonate | 0/5.5 | 0/5.5 |
| Potassium Citrate | 0/16.5 | 0/16.5 |
| Vitamin Mix | 40/10 | 40/10 |
| Choline Bitartrate | 0/2 | 0/2 |
| Total kcal/g | 4056.8 kcal/1055 gm = 3.84 | 4057 kcal/773.8 gm = 5.24 |
| Nutrient composition | Energy supply ratio | |
| Protein | 20% | 20% |
| Carbohydrate | 70% | 20% |
| Fat | 10% | 60% |
