# Supplementary material for: D-Psicose mitigates NAFLD mice induced by a high-fat diet by reducing lipid accumulation, inflammation, and oxidative stress
Source: Front Nutr. 2025 May 27;12:1574151. doi: 10.3389/fnut.2025.1574151 (PMC12148910; doi:10.3389/fnut.2025.1574151)
Supplement: Supplementary file 2 [file Table_2.docx]

**Supplement**

Table S1 The primers of inflammation factors in mouse liver by qRT−PCR.

| Genes | Primer sequence |
| --- | --- |
| TNF-α | F: AACTCCAGGCGGTGCCTATG |
|  | R: TCCAGCTGCTCCTCCACTTG |
| IL-1β | F: CCTGTCCTGCGTGTTGAAAGA |
|  | R: GGGAACTGGGCAGACTCAAA |
| IL-10 | F: GAAGCTCCCTCAGCGAGGACA |
|  | R: TTGGGCCAGTGAGTGAAAGGG |
| *NLRP3* | F: TCTCAAGTCTAAGCACCAACCAG |
|  | R: CGAAGCAGCATTGATGGGAC |
| *Caspase 1* | F: AGGGAATGTGGGACCACATAC |
|  | R: CTGAATCTTTTAACAACACCACTCC |
| *β-actin* | F: AAGCTGTGCTATGTTGCTCTA |
|  | R: GTTTCATGGATGCCACAGGA |

**S2: Western blotting assay**

The target protein in mouse liver tissue was detected using the following procedure. Add an appropriate amount of RIPA (Biosharp, Beijing, China) to the mouse liver tissue and use a homogenizer to fully lyse the tissue to extract total protein. Utilize the BCA method (Biosharp, Beijing, China) to measure the protein concentration in the sample.Standardize the protein content, conduct SDS-PAGE for separation, and transfer to a 0.45 μm PVDF membrane (Thermo Fisher Scientific, Waltham, MA, USA). Block the PVDF membrane with 1% bovine serum albumin for 1 hour, then incubate with antibodies: NF-κB p65 (1:1000, Boster, Wuhan, China), p-p65 (1:500, Proteintech, Wuhan, China), anti-RAGE (1:1000, Proteintech, Wuhan, China), and GAPDH (1:10000, Boster, Wuhan, China). Following incubation, rinse thoroughly with TBST buffer and then incubate with goat anti-rabbit IgG-HRP (1:15000, Boster, China) for 1 hour. Employ an ECL chemiluminescence kit (Millipore, Boston, MA, USA) for exposure, followed by analysis and quantification of the target protein expression using Image J software.

**S3: Histological analysis of mouse liver tissue using HE and Oil Red O staining**

A portion of the mouse livers was fixed with 4% paraformaldehyde and used for paraffin embedding and sectioning. The prepared paraffin sections underwent the steps of deparaffinization and hydration, hematoxylin staining, differentiation, dehydration and clearing, eosin staining, dehydration and clearing, and neutral resin mounting to obtain HE-stained sections, which were used to observe morphological changes in liver tissue. Fresh liver tissue was used to prepare frozen sections, fixed with 4% formaldehyde-phosphate buffer (pH 7.4) for 10 minutes. The sections were washed with water and 60% isopropanol, then stained with 0.4% Oil Red O solution for 10 minutes. The sections were counterstained with hematoxylin after washing with water and 60% isopropanol, then examined microscopically to evaluate lipid deposition in liver tissue.

**S4 Mouse colon contents microbial 16S rRNA detection analysis**

Genomic DNA was extracted from the mouse colon contents for 16S rRNA gene amplification and sequencing. The V3-V4 variable region of the bacterial 16S rRNA gene was PCR amplified using universal primers. The primer sequences were 341F: (5’-CCTACGGGNGGCWGCAG-3’) and 806R (5’-GGACTACHVGGGTATCTAAT-3’). After purification of the amplification products, high-throughput sequencing was performed using the Illumina sequencing platform (NovaSeq). The obtained data were denoised and species annotation was conducted based on the SILVA database, with taxonomic assignment using QIIME2. Further microbial community analysis was performed, including α diversity to assess species richness within samples, β diversity to compare community structure differences between samples, and LEfSe analysis to identify differential microbes between groups.
